# Validity of gait parameters for hip flexor contracture in patients with cerebral palsy

**DOI:** 10.1186/1743-0003-8-4

**Published:** 2011-01-23

**Authors:** Sun Jong Choi, Chin Youb Chung, Kyoung Min Lee, Dae Gyu Kwon, Sang Hyeong Lee, Moon Soek Park

**Affiliations:** 1 Department of Orthopedic Surgery, Synergy Hospital, 115-17 Nonhyun-Dong, Kangnam-Gu, Seoul, 135-010, Republic of Korea; 2 Department of Orthopedic Surgery, Seoul National University Bundang Hospital, 300 Gumi-Dong, Bundang-Gu,Sungnam, Kyungki 463-707, Republic of Korea; 3 Department of Orthopedic Surgery, Dongguk University Ilsan Hospital, 814 Siksa-Dong, Ilsandong-Gu, Koyang, Kyungki 410-773, Republic of Korea

## Abstract

**Background:**

Psoas contracture is known to cause abnormal hip motion in patients with cerebral palsy. The authors investigated the clinical relevance of hip kinematic and kinetic parameters, and 3D modeled psoas length in terms of discriminant validty, convergent validity, and responsiveness.

**Methods:**

Twenty-four patients with cerebral palsy (mean age 6.9 years) and 28 normal children (mean age 7.6 years) were included. Kinematic and kinetic data were obtained by three dimensional gait analysis, and psoas lengths were determined using a musculoskeletal modeling technique. Validity of the hip parameters were evaluated.

**Results:**

In discriminant validity, maximum psoas length (effect size r = 0.740), maximum pelvic tilt (0.710), maximum hip flexion in late swing (0.728), maximum hip extension in stance (0.743), and hip flexor index (0.792) showed favorable discriminant ability between the normal controls and the patients. In convergent validity, maximum psoas length was not significantly correlated with maximum hip extension in stance in control group whereas it was correlated with maximum hip extension in stance (r = -0.933, p < 0.001) in the patients group. In responsiveness, maximum pelvic tilt (p = 0.008), maximum hip extension in stance (p = 0.001), maximum psoas length (p < 0.001), and hip flexor index (p < 0.001) showed significant improvement post-operatively.

**Conclusions:**

Maximum pelvic tilt, maximum psoas length, hip flexor index, and maximum hip extension in stance were found to be clinically relevant parameters in evaluating hip flexor contracture.

## Background

Hip flexion deformity or spasticity is a cause of the abnormal gait observed in cerebral palsy patients. Hip flexor spasticity was reported to cause dynamic restriction of hip extension in the terminal stance and become fixed hip flexion contracture with age in those patients [1-3]. The psoas muscle is a primary cause of hip flexion contracture [4,5] and has been known to be associated with increased anterior pelvic tilt, crouch gait, hip instability and lumbar lordosis, which can eventually cause spondylosis and back pain [1,4,6-8]. The psoas muscle plays an important role in advancing the lower leg during normal gait [4], whereas the dysphasic activity of the hip flexor muscle opposes and limits hip extension in patients with cerebral palsy [4,9-11], which reduces the stride length and gait efficacy.

Despite the role of this muscle in the pathologic gait, the surgical indications of psoas lengthening are somewhat vague. Furthermore, although several kinematic and kinetic variables were shown to represent hip motion during gait and those variables were used to report changes after single event multilevel surgery in patients with cerebral palsy, the clinical relevance of those variables measuring the hip flexor function is unclear.

After 3D modeled muscle length calculated from kinematic data of gait analysis was devised, it was believed that this could be especially useful in measuring dynamic length of multijoint muscle during gait because reflecting the multijoint movement is not easy to follow [12]. Several studies have investigated 3D modeled psoas length [13-15], but its clinical relevance has not been sufficiently verified.

The kinematic and kinetic data of hip motion as well as the 3D psoas length need to be evaluated accurately for clinical use. This study examined the validity of kinematic and kinetic variables measuring the hip flexor function and the 3D modeled psoas length by 1) discriminating the pathologic gait from the normal gait (discriminant validity), 2) correlating those variables (convergent validity), and 3) analyzing post-operative changes (responsiveness).

## Methods

### Inclusion/Exclusion Criteria

This retrospective study was performed at a tertiary referral center for cerebral palsy and was approved by the institutional review board. The study was designed to include a group of normal children and a group of patients with cerebral palsy. For the group of normal children, volunteers aged from 5 to 15 years old were recruited. The exclusion criteria were known neuromuscular disease and an abnormality of lower limb alignment. For the study group, patient selection was based on the medical records since 1997. In order to have a homogenous group of the patients with cerebral palsy, the following inclusion criteria were used: 1) ambulatory patients with spastic diplegia (GMFCS level I-II, gross motor function classification system [16], who had the representative gait pattern consisting of a jump gait pattern [17] with intoeing, equinus, stiff knee, and femoral antetorsion, which is one of the most representative gait patterns of diplega; 2) patients who underwent bilateral single event multilevel surgery (bilateral tendo-Achilles lengthening, distal hamstring lengthening, rectus femoris transfer, femoral derotational osteotomy); 3) a follow-up period of more than one year; 4) the pre-operative and post-operative gait analysis; and 5) 5-15 years of age. The exclusion criteria were patients with a history of gait corrective surgery or selective dorsal rhizotomy, neuromuscular diseases other than cerebral palsy, an asymmetrical gait pattern and surgical procedures other than the index procedures. The demographic data, physical examination (including Thomas test [18]), and gait parameters of the patients, including gender, age, GMFCS level, cadence, step length, and walking speed, were collected. Informed consent for the retrospective review of the gait analysis data of patients and control group was waived by the institutional review board at our hospital.

### Kinematic and kinetic data

The gait analysis laboratory was equipped with a Vicon 370 (Oxford Metrix, Oxford, UK) system consisting of seven CCD cameras and two force plates. Motion was captured while the subjects walked barefoot on a nine-meter walkway, and the kinematic and kinetic data were obtained, which were averaged by three trials. The hip flexion and extension, hip rotation, and pelvic tilt were the key kinematic variables. The kinetic data including time of crossover in the hip flexion-extension moment and the power burst of hip flexor in the late stance were obtained. The hip flexor index was calculated from the kinematic and kinetic data of the hip and pelvic motion, which were maximum pelvic tilt, pelvic tilt range, maximum hip extension in stance, and late stance power burst of hip joint (H3) [19].

### 3D modeled psoas length

**Figure 1:**
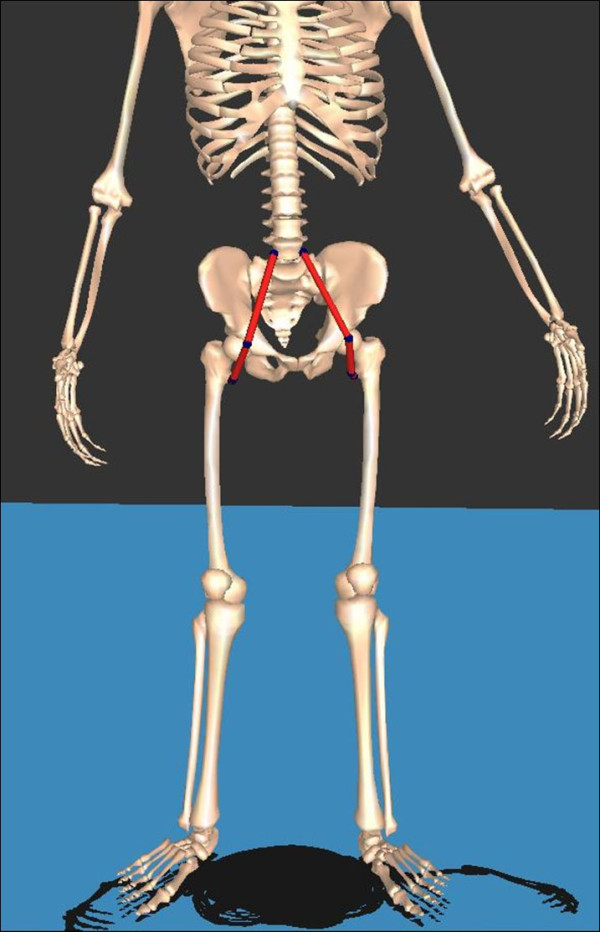
**Three dimensional musculoskeletal modeling image depicting the psoas muscles between their bony origins and insertions with the knee and hip joint in 0° of extension, which represents static psoas length**.

**Figure 2:**
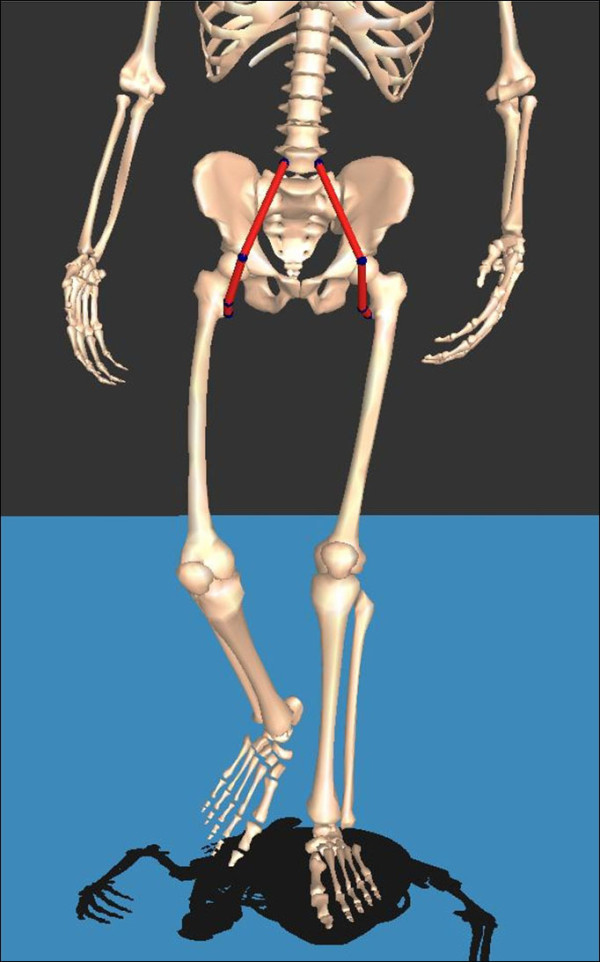
**Psoas length (distance between its bony origin and insertion) changed throughout the gait cycle, which is dynamic psoas length**.

**Figure 3:**
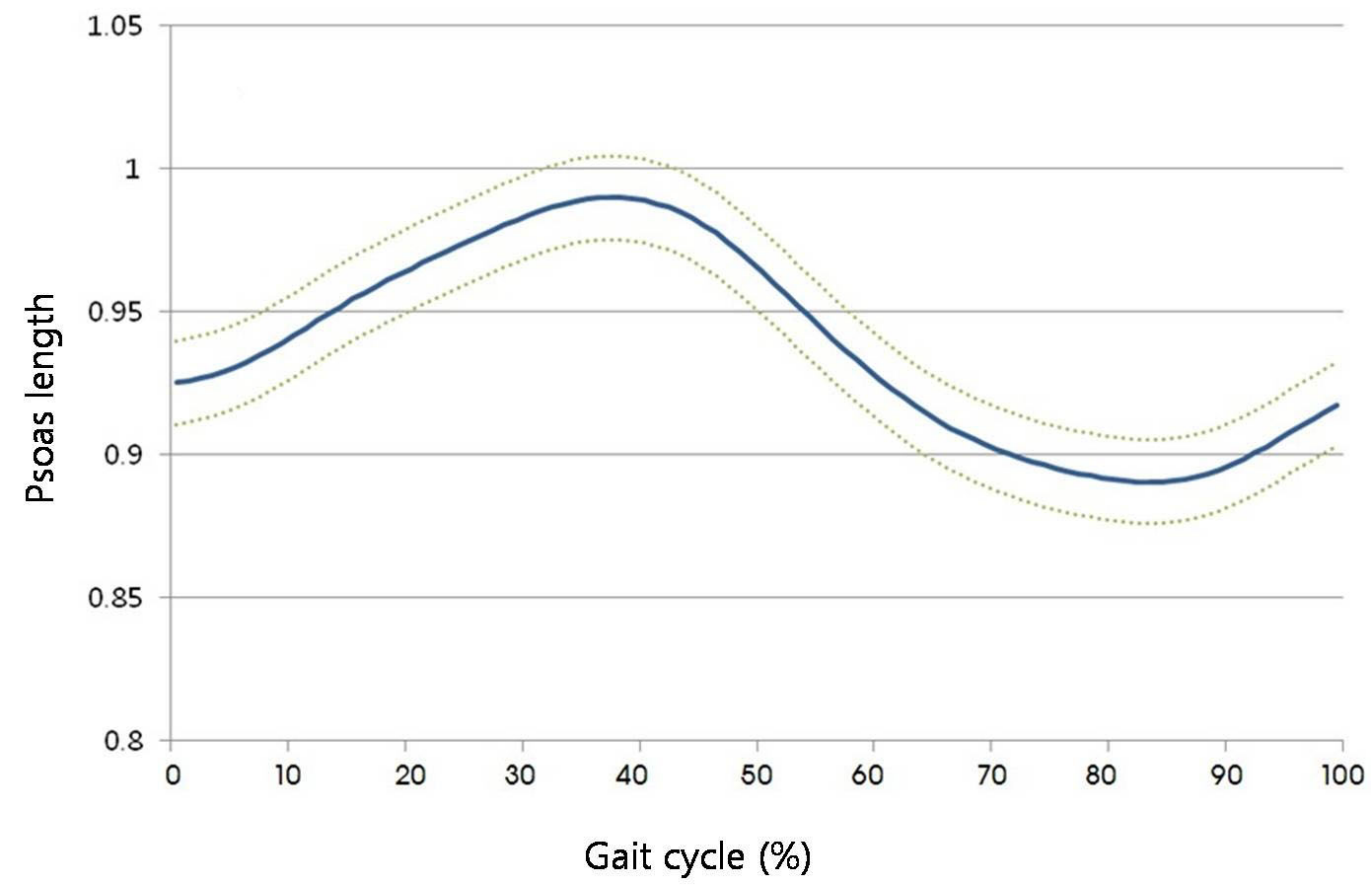
**Standardized psoas length was calculated and depicted throughout the gait cycle, which is dynamic psoas length divided by static psoas length**.

The psoas length was obtained using interactive musculoskeletal modeling [20] software (SIMM, Motion Analysis Corporation, Santa Rosa, CA) (Figures 1 and 2). The psoas length was determined to be between the muscular origin and insertion, which were the transverse process of the lumbar spine and lesser trochanter of the femur, respectively. However, in this study, spine motion was not included. Calculated average psoas origin was used, and calculated average pelvic brim was used as via points. The anatomic points were calculated from the kinematic data of femur and pelvis. Although psoas is a multijoint muscle, only hip angles were reflected in its length. The psoas length was standardized by dividing the calculated psoas length during gait by the muscle length when the subjects were in a simulated anatomic position. This standardized psoas length was recorded continuously during the gait cycle (Figure 3) and included for analysis.

### Validity of kinematic and kinetic variables, and psoas length in hip flexor function

There are no gold standards for measuring hip flexor function during gait. Therefore, the validity of kinematic and kinetic data regarding hip flexor function relies on the content validity and construct validity. Construct validity is comprised of the discriminant validity and convergent validity. The discriminant validity [21] is one facet of the construct validity, and reflects the degree to which an instrument can distinguish between or among different concepts or constructs [22]. This is the ability to detect clinically relevant difference. In this study, effect-size r [23] between the normal control and the patient groups were assessed as in previous studies [24-27]. Convergent validity [21,28] which is another type of construct validity, occurs when the scales of a measurement correlate as expected with the related scales of another measurement. In this study, the 3D modeled psoas lengths were compared with the kinematic and kinetic hip parameters representing hip and pelvic motion. Responsiveness [29] was tested by comparing the pre-operative and post-operative variables.

### Statistical Analysis

One of the principal variables in this study was the psoas length on which we had few previous studies that we could refer to. We assumed that 1% of difference in psoas length between the control and patient groups would be clinically relevant, and prior power analysis (alpha error 0.05, power 0.8) revealed that over 17 subjects would be needed on each group. The average of the variables of right and left legs were used for data analysis to ensure data independence.

Statistical analysis was performed using SPSS Ver. 15.0 (SPSS, Chicago, Illinois). The normal distribution of the data was tested using a Kolmogorov-Smirnov test. The discriminant validity was assessed by the effect-size r [23] for the kinematic and kinetic variables and psoas length. The Effect size is a name given to a family of indices that measures the magnitude of a certain effect and is generally measured in two ways: as the standardized difference between two means, or as the correlation between the independent variable classification and individual scores on the dependent variable. This correlation is called the effect size correlation (effect-size r) and was used for the discriminant validity in this study. Correlations between each of the kinematic and kinetic variables and psoas length were analyzed using a Pearson's correlation test for convergent validity. The comparison of the data between the patients and normal controls was performed using a t-test, and the post-operative changes in the patients were analyzed using a paired t-test. A p value < 0.05 was considered significant. For multiple testing, statistical significance was adjusted for family wise error.

## Results

Twenty-four patients with cerebral palsy were finally included in this study. The mean age of the patients was 6.9 years (SD 1.6 years), and there were 15 males and 9 females. The GMFCS levels were I in 15 patients and II in 9 patients. The mean age of the 28 normal controls was 7.6 years (SD 2.4 years), and there were 17 males and 11 females. The mean age and gender ratio were not significantly different between the two groups (p = 0.222 and p = 0.973) (Table 1).

**Table 1:** Demographic data and gait parameters

	Patients	Normal controls	*p*
N	24	28	
Age (years)	6.9 (1.6)	7.6 (2.4)	0.222
Sex (M:F)	15:9	17:11	0.973
Follow up period (years)	1.1 (0.2)	-	
GMFCS level (I/II)	15/9	-	
Gait parameters			
Cadence (No./min)	101.2 (14.2)	112.5 (12.9)	0.001
Step length (cm)	35.3 (6.2)	53.0 (9.2)	<0.001
Walking speed (cm/s)	59.9 (13.5)	99.9 (16.9)	<0.001

Data are presented as mean (SD).

### Discriminant validity of kinematic and kinetic data, and psoas length

The discriminant validity between the patients and normal control group was highest in hip flexor index (effect size r = 0.792) followed by maximum hip extension in stance (0.743), maximum psoas length (0.740), maximum hip flexion in late swing (0.728) and maximum pelvic tilt (0.710). Kinetic data, including the time of crossover in hip flexion-extension moment (0.059) and power burst of hip flexor in late stance (0.020), showed an unsatisfactory discriminant validity (Table 2).

**Table 2:** Discriminant validity of hip parameters

	Cerebral palsy	Normal controls	*p*	Effect size (r)
**Thomas test (°)**	7.4 (6.8)	0.6 (1.9)	<0.001	0.561
**Pelvic tilt (°)**				
maximum	21.9 (4.9)	12.1 (4.8)	<0.001	0.710
minimum	12.6 (5.9)	6.9 (3.9)	0.008	0.497
range	9.4 (3.5)	5.2 (2.5)	0.002	0.562
mean	17.5 (5.1)	9.5 (4.1)	<0.001	0.654
**Max hip extension in stance (°)**	-0.5 (6.1)	11.1 (4.2)	<0.001	0.743
**Max hip flexion in late swing (°)**	50.4 (5.9)	38.2 (5.5)	<0.001	0.728
**Hip rotation (°)**				
maximum	12.8 (8.2)	12.8 (9.9)	0.997	0.005
minimum	0.2 (9.2)	-11.8 (13.2)	0.005	0.467
range	12.6 (4.1)	24.7 (11.5)	0.009	0.573
mean	6.3 (8.9)	0.1 (10.0)	0.086	0.308
**Psoas length (%)**				
maximum	99.2 (1.3)	101.5 (0.8)	<0.001	0.740
minimum	87.5 (1.4)	90.4 (1.7)	<0.001	0.684
range	11.7 (1.5)	11.1 (1.2)	0.126	0.212
mean	93.6 (1.3)	96.0 (1.3)	<0.001	0.676
**TOC (%)**	28.2 (11.5)	27.0 (8.6)	0.767	0.059
**H3 (W/kg)**	0.3 (0.4)	0.3 (0.4)	0.920	0.020
**HFI**	5.9 (1.4)	1.9 (1.7)	<0.001	0.792

TOC, time of cross over in hip flexion/extension moment; H3, late swing power burst in hip joint flexion/extension power; HFI, hip flexor index.

Data are presented as mean (SD).

### Convergent validity of kinematic and kinetic data, and psoas length

In the normal control group, the correlation coefficient between the maximum psoas length and maximum hip extension in stance was -0.420 (p = 0.065). The maximum psoas length showed correlation coefficients of 0.601, -0.651, and -0.448 with the step length, time of crossover in hip flexion-extension moment, and hip flexor index, respectively. The minimum psoas length showed no significant correlation with the kinematic and kinetic variables (Table 3).

**Table 3:** Correlation coefficients between psoas length and gait parameters in control group

	Max PL	Min PL	Range PL	Mean PL
**Thomas test (°)**	-0.253	-0.416*	0.407*	-0.450*
**Pelvic tilt (°)**				
maximum	-0.576*	-0.206	0.050	-0.399
minimum	-0.595*	-0.141	-0.018	-0.296
range	-0.110	-0.140	0.108	-0.240
mean	-0.672*	-0.226	0.044	-0.420
**Max hip extension in stance (°)**	-0.420	-0.029	-0.082	-0.201
**Max hip flexion in late swing (°)**	-0.312	-0.327	0.238	-0.302
**Hip rotation (°)**				
maximum	0.140	-0.098	0.133	-0.172
minimum	-0.316	-0.101	0.015	-0.233
range	0.532*	0.012	0.128	0.088
mean	-0.128	-0.091	0.055	-0.226
**TOC (%)**	-0.651*	-0.085	-0.107	-0.344
**H3 (W/kg)**	0.140	-0.305	0.326	-0.234
**HFI**	-0.448*	-0.081	-0.039	-0.269
**Cadence (No./min)**	-0.278	-0.208	0.131	-0.288
**Step length (cm)**	0.601*	0.206	-0.044	0.355
**Walking speed (cm/s)**	0.511*	0.149	-0.011	0.232

TOC, time of cross over in hip flexion/extension moment; H3, late swing power burst in hip joint flexion/extension power; HFI, hip flexor index; *, p < 0.05.

In the patients group, the maximum psoas length showed a significant correlation with the maximum hip extension in stance (r = -0.933, p < 0.001). The correlation coefficient between the maximum psoas length and hip flexor index was -0.467 (p = 0.001). There was no significant correlation between the maximum psoas length and step length (Table 4). Thomas test did not show significant correlation with maximum psoas length in control and patient groups.

**Table 4:** Correlation coefficients between psoas length and gait parameters in patients group

	Max PL	Min PL	Range PL	Mean PL
**Thomas test (°)**	-0.116	0.408*	-0.476*	0.200
**Pelvic tilt (°)**				
maximum	-0.331*	-0.611*	0.326*	-0.610*
minimum	-0.446*	-0.474*	0.109	-0.547*
range	0.286*	-0.054	0.268	0.069
mean	-0.457*	-0.560*	0.182	-0.635*
**Max hip extension in stance (°)**	-0.933*	-0.299*	-0.427*	-0.747*
**Max hip flexion in late swing (°)**	-0.137	-0.740*	0.596*	-0.585*
**Hip rotation (°)**				
maximum	-0.367*	-0.445*	0.142	-0.495*
minimum	-0.388*	-0.423*	0.105	-0.442*
range	0.098	-0.003	0.077	-0.072
mean	-0.369*	-0.421*	0.117	-0.450*
**TOC (%)**	-0.324*	-0.183	-0.090	-0.417*
**H3 (W/kg)**	0.135	0.004	0.106	0.009
**HFI**	-0.467*	-0.503*	0.120	-0.646*
**Cadence (No./min)**	-0.133	-0.001	-0.100	-0.109
**Step length (cm)**	0.101	-0.074	0.147	-0.122
**Walking speed (cm/s)**	0.040	-0.022	0.051	-0.122

TOC, time of cross over in hip flexion/extension moment; H3, late swing power burst in hip joint flexion/extension power; HFI, hip flexor index; *, p < 0.05.

### Responsiveness of kinematic and kinetic data, and psoas length

The maximum pelvic tilt, maximum hip extension in stance, maximum psoas length and hip flexor index showed significant improvement after surgery (p = 0.008, p = 0.001, p < 0.001, and p < 0.001 respectively). There was no significant post-operative change in the range of psoas lengths (p = 0.158) and power burst of the hip flexor in late stance (p = 0.627) (Table 5).

**Table 5:** Responsiveness of psoas length and gait parameters in patients with spastic diplegia

	Pre-operative	Post-operative	*p*
**Pelvic tilt (°)**			
maximum	21.9 (4.9)	18.8 (4.9)	0.008
minimum	12.6 (5.9)	12.9 (5.0)	0.897
range	9.4 (3.5)	5.9 (2.1)	<0.001
mean	17.5 (5.1)	15.9 (4.9)	0.126
**Max hip extension in stance (°)**	-0.5 (6.1)	4.6 (7.5)	0.001
**Max hip flexion in late swing (°)**	50.4 (5.9)	42.9 (5.1)	<0.001
**Hip rotation (°)**			
maximum	12.8 (8.2)	10.0 (4.9)	0.107
minimum	0.2 (9.2)	-5.2 (7.0)	0.016
range	12.6 (4.1)	15.3 (4.6)	0.015
mean	6.3 (8.9)	2.3 (5.8)	0.051
**Psoas length (%)**			
maximum	99.2 (1.3)	100.3 (1.2)	<0.001
minimum	87.5 (1.4)	89.3 (1.6)	<0.001
range	11.7 (1.5)	11.1 (1.9)	0.158
mean	93.6 (1.3)	95.0 (1.2)	<0.001
**TOC (%)**	28.2 (11.5)	24.6 (12.3)	0.173
**H3 (W/kg)**	0.3 (0.4)	0.5 (2.0)	0.627
**HFI**	5.9 (1.4)	3.8 (2.0)	<0.001
**Cadence (No./min)**	101.2 (14.2)	103.0 (16.6)	0.251
**Step length (cm)**	35.3 (6.2)	41.1 (6.2)	<0.001
**Walking speed (cm/s)**	59.9 (13.5)	71.1 (15.8)	<0.001

TOC, time of cross over in hip flexion/extension moment; H3, late swing power burst in hip joint flexion/extension power; HFI, hip flexor index.

Data are presented as mean (SD).

All patients underwent bilateral femoral derotation osteotomy, rectus femoris transfer, distal hamstring lengthening, and tendo-Achilles lengthening as single event multilevel surgery.

## Discussion

The patients with cerebral palsy showed a shorter psoas length and smaller maximum hip extension in stance than the normal control group. The maximum psoas length was found to reflect the kinetic and kinematic data of hip motion. The hip flexor index showed satisfactory discriminant and convergent validity, showing a significant correlation with the psoas length. The result of the cross correlation revealed an excellent correlation between the maximum psoas length and maximum hip extension in the patients group (Table 6).

**Table 6:** Validity of the parameters for hip flexor contracture

	Discriminant validity (effect-size r)	Convergent validity in normal control (correlation r)	Convergent validity in CP (correlation r)	Responsiveness (effect-size r)
Maximum pelvic tilt (°)	****	***	**	**
Max hip extension in stance (°)	****	***	*****	**
HFI	****	***	***	***
Maximum psoas length (%)	****	†	†	***

HFI, hip flexor index.

*, 0-0.2; **, 0.2-0.4; ***, 0.4-0.6; ****, 0.6-0.8; *****, 0.8-1.0.

†, convergent validity was the correlation coefficient with maximum psoas length.

The patients with cerebral palsy showed a shorter maximum psoas length, larger pelvic tilt, and more sagittal pelvic motion than the normal control group. The maximum hip extension in stance was limited in the patient group, which was possibly caused by a shorter psoas length. However, the range of psoas lengths was similar in the patients and control group suggesting that muscle excursion was not significantly different. The kinetic variable, including the time of crossover in the hip flexion-extension moment and the power burst of the hip flexor in late stance, were similar in the patients and controls. In this study, the maximum psoas length, hip flexor index and sagittal pelvic motion showed favorable discriminant validity. The correlation coefficient between the maximum psoas length and maximum hip extension in stance was -0.420 (p= 0.065) in the control group whereas it was -0.933 in the patients (p < 0.001). The shortened maximum psoas length in the patients appeared to limit the maximum extension of the hip joint. However, the maximum psoas length might not have been the limiting factor in maximum hip extension in the control group. This could have cause different correlation coefficients of the two groups between maximum psoas length and maximum hip extension in stance.

It has been reported that the psoas length could be confounded by the femoral anteversion [30], which is supported by the results of this study. The psoas length increased post-operatively, even though no psoas procedures had been performed in addition to femoral derotation osteotomy. Therefore, femoral derotation osteotomy may improve the dynamic psoas length possibly by moving the lesser trochanter forward. However, this requires further examination.

This study had some limitations. First, although small changes in the kinetic and kinematic variables in the study were statistically significant, they may have been due to marker placement variability, despite this being performed by a single experienced operator. Second, the 3D psoas length did not reflect the lumbar spinal motion which could affect the real psoas length significantly because the model did not contain the trunk marker sets. Therefore, the 3D modeled psoas length might not be as accurate as expected.

## Conclusions

The patients with cerebral palsy showed a shorter psoas length than the normal control group. The hip flexor index and psoas length showed good discriminant validity. There was an excellent correlation between the maximum psoas length and maximum hip extension in the patients group. There was evidence that estimated psoas length could be improved after femoral derotation osteotomy, even though no psoas procedure had been performed.

## Competing interests

The authors declare that they have no competing interests.

## Authors' contributions

CYC, MSP, and KML have made substantial contributions to conception and design. SJC, DGK, and SHL have been involved in acquisition of data, analysis and interpretation of data. SJC, KML and MSP drafted the manuscript. All authors read and approved the manuscript.
